# Secretory Carcinoma of the Breast: Report of Two Cases and Review of the Literature

**DOI:** 10.1155/2015/581892

**Published:** 2015-07-05

**Authors:** Vivekanand Sharma, Gajendra Anuragi, Suresh Singh, Pinakin Patel, Arpita Jindal, Raj Govind Sharma

**Affiliations:** ^1^Division of Surgical Oncology, Department of Surgery, S.M.S. Medical College and Hospital, Jaipur, Rajasthan 302004, India; ^2^Department of Pathology, S.M.S. Medical College and Hospital, Jaipur, Rajasthan 302004, India

## Abstract

Secretory carcinoma of the breast is an extremely rare subtype of breast cancer characterized by intracellular or extracellular secretion and granular eosinophilic cytoplasm of the neoplastic cells. The disease which was considered to be predominant in younger age group has been recognized in adult population too and tends to show slow growth and indolent behavior. The disease occurs preferentially in females and only 27 cases have been reported amongst males. An optimal treatment for the disease subtype has been debated because of the paucity of data. We report two cases (one female and one male) of this rare disease that underwent treatment at our institution.

## 1. Introduction

Secretory breast carcinoma (SBC) was first described by McDivitt and Stewart [1]. It is a very rare subtype of breast cancer and comprises less than 0.15% of invasive breast cancers [2]. It is identified by its distinct histomorphology and usually is associated with a favourable prognosis. Secretory carcinoma was first reported in children and the older literature identified the disease by the name of juvenile breast carcinoma. However, after the review by Tavassoli and Norris [3], it was recognised that it may occur in adult patients as well. Recently, the tumor was found to be associated with a distinct ETV6-NTRK3 mutation which confers the tumor proliferative and survival advantage.

## 2. Clinical Case Report 1

A 55-year-old lady with no family history of breast cancer presented to our department in January 2014. The patient had noticed a lump in the right breast a month back which had gradually grown. She underwent a lumpectomy for the same at another center and was referred with the histopathology suggestive of secretory carcinoma.

Clinical examination showed a 5 cm long healed scar in the upper outer quadrant of the right breast. The nipple and areola appeared normal. No clinical alterations in either the ipsilateral or contralateral axillae were found. There was no evidence of metastatic disease on evaluation.

**Figure 1:**
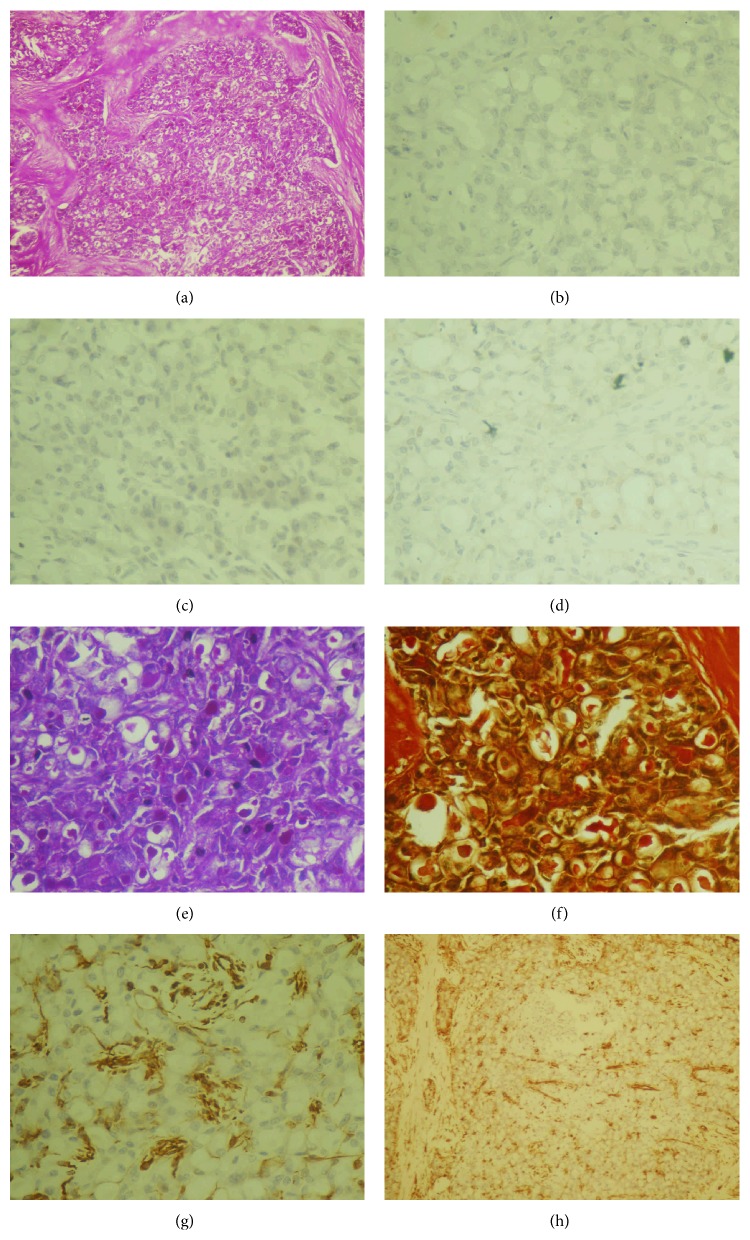
(a) Scanner view of hematoxylin and eosin stained slide. (b), (c), (d) Immunohistochemistry for estrogen receptor (b), progesterone receptor (c), and HER 2neu (d). Special stains immunohistochemistry showing PAS staining (e), mucicarmine (f), and vimentin at 400x (g) and at 100x (h).

The biopsy of the lumpectomy specimen showed a 3 × 2 cm mass harbouring microscopically an intraductal neoplasm with the tumor cells showing glands and microcystic spaces showing abundant pale secretions. The cells had abundant pale staining cytoplasm with small round low grade nuclei. There was no perineural or vascular invasion. The biopsy hence suggested a secretory carcinoma of the breast (Figure 1). Immunohistochemistry of the tumor on review of paraffin embedded blocks showed neoplastic cells with triple negative results (estrogen receptor and progesterone receptor and Her-2/neu protein expression negative). The tumor also stained positive for PAS, mucicarmine, and vimentin (Figure 1).

The patient opted for a modified radical mastectomy with axillary dissection citing difficulty to routinely follow-up for radiotherapy. On subsequent histopathological examination the mastectomy specimen and all 16 lymph nodes resected were negative for the presence of the tumor.

Postoperatively the patient made an uneventful recovery and was treated with 6 cycles of combination of Adriamycin, Cyclophosphamide, and Paclitaxel. The patient is healthy after 15 months of follow-up.

## 3. Case Report 2

A 12-year-old mentally subnormal boy from rural background was brought by his family with a lump in the left subareolar region which was first noticed 6 years back as a small pea sized nodule and grew painlessly to the size of presentation. The patient had no other complaints and his relatives denied any history of similar complaints in the family. The family members reported delayed milestones for the patient who performed poorly for his age and had poor speech and mental development. On examination the patient showed hypertelorism, low set ears, and left sided undescended testis. The breast examination showed a 6 × 6 cm size lump underlying the areola which was firm to hard in consistency and fixed to the overlying thinned skin. The lump was mobile over the underlying muscle. The patient had no axillary lymphadenopathy. On evaluation, otorhinolaryngoscopic evaluation of the patient was within normal limits. Core needle biopsy of the patient showed fibrocollagenous tissue with mild epithelial hyperplasia and was suggestive of gynaecomastia. Ultrasonography of the axillae and the abdomen was normal. Ultrasonography of the scrotum localised the left testis to the left inguinal region.

**Figure 2:**
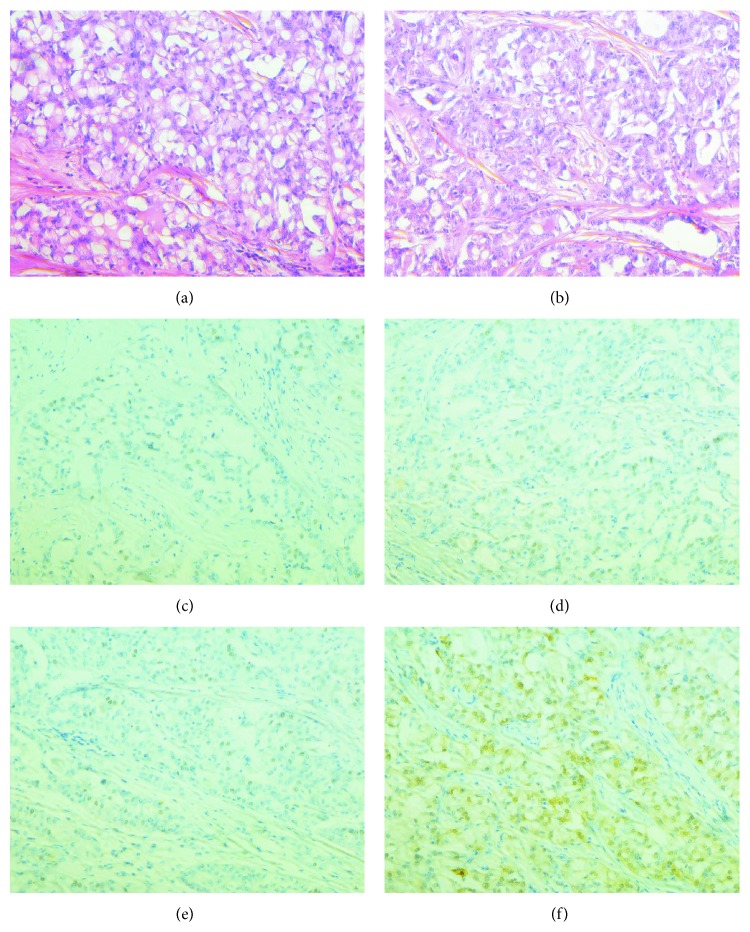
(a), (b) Hematoxylin and eosin showing sheets of glands. Immunohistochemistry for estrogen receptor (c), progesterone receptor (d), and HER 2neu (e) and S-100 positivity (f) each on 400x.

In view of clinical suspicion the patient was advised a wide local excision of the lump and axillary sampling which on histopathological examination revealed a 5 × 4 cm firm grey lesion harbouring secretory carcinoma with foci of stromal fibrosis and apocrine change with pathologically negative 8 lymph nodes. The margins were negative for presence of the tumor. The tumor was negative for estrogen and progesterone receptors and Her2neu. On immunohistochemistry the tumor stained positive for S-100 stain (Figure 2).

In view of the subnormal mentation of the patient and the lack of definitive supportive data in the disease subset, his relatives refused adjuvant therapy. The patient subsequently is on periodic surveillance and is healthy after 6 months of follow-up.

## 4. Discussion

Secretory carcinoma is a very rare type of breast carcinoma. Lamovec and Bracko reported 4 cases of SBC in their retrospective series of 7038 breast carcinoma cases [4], and Botta et al. [5] found one case of SBC among 3000 breast carcinoma cases. Li et al. reported 15 cases in their pathologic review of 10000 breast carcinoma cases [6].

The age at presentation varies from 3 to 87 years old with a mean and median age of presentation of 33 and 40 years, respectively [7]. Most of the cases occur in females with a male : female ratio of 1 : 6. In our review we found only 27 other cases of the disease in male patients [6]. Reports suggest that the disease tends to be more aggressive in males.

The patients commonly present with a slow growing, painless, well-circumscribed, mobile, palpable, subareolar mass in the breast. The mean tumor size is 3 cm. The literature mentions nodal involvement in 15% of the patients at presentation [8]. Although rare, metastatic disease at presentation has also been reported [9].

On ultrasonography a solitary, microlobulated, hypoechoic round or oval or tubular mass is seen resembling a benign lesion or well-circumscribed carcinoma. The typically slow growth and imaging make it a challenging diagnosis. The differential diagnoses include a wide range of benign conditions or malignant lesions (i.e., cystic hypersecretory hyperplasia, juvenile papillomatosis with apocrine metaplasia or mucinous carcinoma, and apocrine carcinoma).

Invasive secretory carcinoma can be differentiated from these entities by demonstration of lack of myoepithelial cell layer on a core needle biopsy. SBCs can present with several histological patterns including solid, microcystic, and ductal, with many tumors containing all three patterns [10]. The tumor cells are polygonal with granular eosinophilic cytoplasm. Atypia is minimal or absent and mitotic activity is low. The two distinctive pathological characteristics of SBC are intracellular/extracellular secretions and granular eosinophilic cytoplasm of the neoplastic cells. The intracellular and extracellular secretions can be periodic acid Schiff (diastase resistant) and mucicarmine positive. Although SBC is well circumscribed macroscopically, there may be foci of invasion in the surrounding breast tissue and associated ductal carcinoma in situ, which can be responsible for local recurrence after incomplete resection. The usefulness of FNA in pediatric patients is established but unproven in adults in whom core needle has been the preferred method.

Immunohistochemistry shows the SBC to be triple negative tumors with recently recognized balanced translocation t(12;15) ETV6-NTRK3 fusion gene. This is the first demonstration of a balanced translocation in breast cancer and was known to be associated with congenital fibrosarcoma and mesoblastic nephroma, two morphologically similar pediatric mesenchymal tumors.

Surgery is considered the mainstay of treatment of secretory carcinoma; however, due to scarcity of reported cases no published guidelines management exists. The demonstration of late local recurrence has led many to propose mastectomy for the patients with this disease [11]. Conservative surgery with delayed reconstruction is particularly lucrative in younger patients where breast development has not occurred. Considering the overall incidence of axillary lymph node infiltration is around 30% in children and adults regardless of gender an axillary lymph node dissection is advocated by some authors for tumors ≥2 cm [12]. Nevertheless, sentinel node biopsy may be useful for secretory carcinomas and recent reports suggest feasibility.

Adjuvant chemotherapy and radiation have been tried for the disease without much success. This falls contrary to the high degree of chemosensitivity shown by congenital fibrosarcomas and mesoblastic nephromas to traditional chemotherapeutic drugs. At present, they are considered mainly for the rare advanced presentation of disease. The identification of the specific gene mutation may pave way for targeted molecular therapies for this disease.

## 5. Conclusion

Secretory breast cancer is a rare indolent tumor that is best treated surgically. Conservative surgery owing to high rate of local recurrence has little role in adults. The role of adjuvant therapy also remains poorly defined and better understanding may allow targeted therapies in the future.
